# Analysis and functional annotation of expressed sequence tags from the fall armyworm *Spodoptera frugiperda*

**DOI:** 10.1186/1471-2164-7-264

**Published:** 2006-10-19

**Authors:** Youping Deng, Yinghua Dong, Venkata Thodima, Rollie J Clem, A Lorena Passarelli

**Affiliations:** 1 Molecular, Cellular, and Developmental Biology Program, Division of Biology, Kansas State University, Manhattan, Kansas 66506, USA; 2 Department of Biological Sciences, The University of Southern Mississippi, Hattiesburg, MS 39402, USA

## Abstract

**Background:**

Little is known about the genome sequences of lepidopteran insects, although this group of insects has been studied extensively in the fields of endocrinology, development, immunity, and pathogen-host interactions. In addition, cell lines derived from *Spodoptera frugiperda *and other lepidopteran insects are routinely used for baculovirus foreign gene expression. This study reports the results of an expressed sequence tag (EST) sequencing project in cells from the lepidopteran insect *S. frugiperda*, the fall armyworm.

**Results:**

We have constructed an EST database using two cDNA libraries from the *S. frugiperda*-derived cell line, SF-21. The database consists of 2,367 ESTs which were assembled into 244 contigs and 951 singlets for a total of 1,195 unique sequences.

**Conclusion:**

*S. frugiperda *is an agriculturally important pest insect and genomic information will be instrumental for establishing initial transcriptional profiling and gene function studies, and for obtaining information about genes manipulated during infections by insect pathogens such as baculoviruses.

## Background

The nucleotide sequences from numerous animal genomes have been derived and include examples from both vertebrate and invertebrate organisms. In addition, determination of the genomic sequences of many more organisms are in progress, yielding a broad picture of the diversity and common pathways among differing organisms. Genome sequences for the insects *Apis mellifera *(honeybee), *Anopheles gambiae *(mosquito), *Drosophila melanogaster *(fruit fly), and *Bombyx mori *(silkworm) have been reported [1-5], and additional insect genome sequences including *Acyrthosiphon pisum *(pea aphid), *Aedes aegypti *and *Culex pipiens *(mosquitoes), several *Drosophila *species, *Nasonia vitripennis *(parasitoid wasp), *Rhodnius prolixus *(insect vector for *Trypanosoma cruzi*), and *Tribolium castaneum *(red flour beetle) are anticipated. In addition, a number of EST databases derived from Lepidoptera are available (NCBI dbEST). The Lepidoptera (moths and butterflies) are the second largest order of the class Insecta. As such, they are a diverse group of insects distributed worldwide and throughout different climates ranging from that in Siberia to the tropics. Thus, it is important to compare the genomes of a number of species within the order and to that of other insects.

Lepidoptera are viewed as being among the most beautiful insects, yet their larvae are major pests to economically important crops and forests. Among the Lepidoptera, the silkworm, *B. mori*, has been studied intensively, since it is a commercially important insect. In addition, the Lepidoptera are valued as models to examine insect-plant and insect-pathogen interactions. Their study and comparative genomic analyses will render valuable tools for insect pest management and the improvement of widely used lepidopteran pathogens, the baculoviruses, as foreign gene expression vectors.

In this study, we report the establishment of an expressed sequence tag (EST) database of 1,195 unique sequences from the cell line IPLB-SF-21 (SF-21) [6], a cell line derived from immature ovaries of pupae of the fall armyworm, *Spodoptera frugiperda*. This and other EST databases can serve as the starting point from which other *S. frugiperda *genome clones can be surveyed or to explore gene expression profiles using microarray assays. More importantly, these and additional ESTs can be the basis for comparative genomic analyses among Lepidoptera or other genomes within the Insecta.

## Results

### General sequence survey

**Figure 1:**
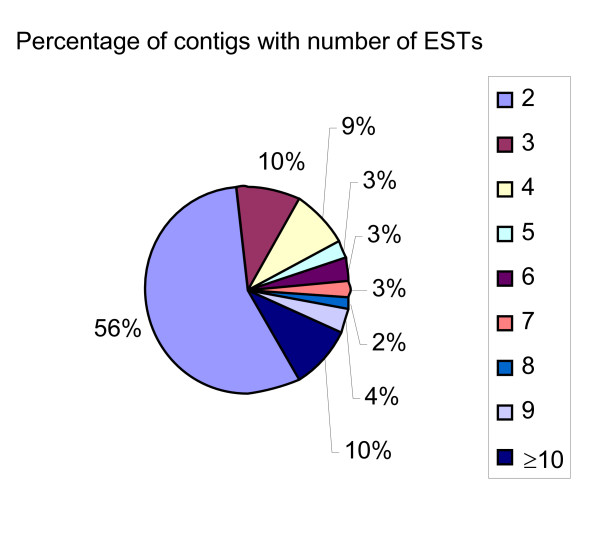
**Distribution of *S. frugiperda *ESTs**. Percentage distribution of contig sequences with number of ESTs. The color-coded legend indicates the number of ESTs in the contig sequences.

To start characterizing SF-21 sequences, two independent directionally cloned cDNA libraries that had been previously constructed for use in yeast two-hybrid screens using the plasmid vectors pB42AD and pYES2 (Invitrogen) were utilized. Initially, about 200 clones were partially sequenced from each library to assess library quality. Both libraries yielded acceptable results. The library cloned in pYES2 was selected for further sequencing. In all, a total of 3,365 cloned inserts were subjected to single-pass sequencing from their 5' ends, 192 clones in the vector pB42AD and 3,173 clones in the vector pYES2. The 3,365 sequences were trimmed of vector sequences, poly A/T tails, low quality, adaptor, and contaminating bacterial sequences, and screened for a minimum length of 200 bp. This resulted in a total of 2,367 high quality ESTs with an average length of 610 bases (156 sequences from pB42AD and 2,211 sequences from pYES2). No attempt was made to carry out sequencing to saturation. These ESTs were assembled using the CAP3 [7] program and verified using the Phrap [8] program. Both programs assemble overlapping ESTs to commence forming contigs. A total of 1,417 ESTs were assembled into 244 contigs, leaving 951 sequences as singlets. Contigs and singlets together resulted in 1,195 unique sequences that putatively represent different transcripts. The number of ESTs in the 244 contigs varied from 2 to 63; 56% of contig sequences had two ESTs, 10% had three ESTs, and 10% had greater than 10 ESTs (Fig. 1). The average length of the assembled contigs was 854 bases; longer than the average length of singlets (617 bases). The longest contig sequence, contig 138, was 2,361 bases.

### Highly redundant genes

A total of 14 contigs containing 307 ESTs were highly redundant. This accounted for more than 13% of the total high quality ESTs. The minimum number of ESTs that made up these most highly redundant contigs was 13 (Table 1). Distribution of ESTs in each contig can be accessed via the ESTMD database [9] using the contig viewer search function. The best matched genes of 6 contigs, totaling 138 ESTs, are from *S. frugiperda*, verifying the source of the cDNAs. Nearly half (6) of the highly redundant contigs, totaling 90 ESTs, had significant homology to various ribosomal proteins, indicating high transcript abundance of ribosomal protein genes, as expected. Four contigs totaling 89 ESTs had matches similar to sequences derived from mitochondrial cytochrome b or cytochrome oxidase subunits (Table 1). The most redundant contig was composed of 63 ESTs and had significant homology to NADH dehydrogenase subunit -1 (ND-1) from *S. frugiperda *[10].

### Comparative sequence analysis of *S. frugiperda *cDNA data

We used the 1,195 unique ESTs to search non-redundant protein databases using BLASTX (Table 2). A total of 724 sequences (60.6%) matched known proteins at a cut-off expectation (E)-value of 10^-5 ^or below. Eleven sequences (0.9%) had hits with E-values at E < 10^-150^, 53 sequences (4.4%) had hits with E-values between 10^-150 ^and 10^-100^, 283 sequences (23.6%) had hits with E-values between 10^-100 ^and 10^-50^, 237 sequences (19.8%) had hits with E-values between 10^-50 ^and 10^-20^, and 140 sequences (12%) had hits with E-values between 10^-20 ^and 10^-5^. The main matched E-value, between 10^-50 ^and 10^-20^, included 510 searched unique sequences, which was more than 70% of the 724 matched sequences. The remainder of the unique sequences (39.4%) had no meaningful matches (E > 10^-5^).

Given that *Drosophila *is the most thoroughly annotated insect genome [11], we compared *S. frugiperda *unique sequences with *Drosophila *genes using BLASTX. A total of 677 sequences had hits with *Drosophila *genes at E < 10^-5^, that is, 56.7% of the 1,195 unique sequences (Table 2). A subset of 53 unique sequences (4.4%) matched to *Drosophila *genes with the cut-off equal to E < 10^-100^. A total of 274 sequences (22.9%) had matches with *Drosophila *genes between 10^-100 ^and 10^-50^, 224 sequences (18.7%) had matches between E-values of 10^-50 ^and 10^-20^, and 116 sequences (9.7%) had matches between E-values of 10^-20 ^and 10^-5^(Table 2).

We compared our unique sequences from the SF-21 cell line with ESTs obtained from another *S. frugiperda*-derived cell line, Sf9 [12], using BLASTN [13]. A total of 419 sequences (35%) matched the ESTs from Sf9 cells with an E-value equal to 0 (Table 5). A total of 241 sequences (20.2%) were similar but not exact matches with ESTs from Sf9 with E-values > 0 and < 10^-5^. In addition, almost half of our ESTs (535 sequences or 44.8%) had no significant match with the Sf9 ESTs. Therefore, 776 of our sequences (65%) were not previously reported in the Sf9 EST project.

We also compared our sequences with about 4,000 *S. frugiperda *midgut-specific ESTs available in NCBI dbEST database using BLASTN. Only 88 sequences (7.36%) matched with E-value equal to 0 (Table 5). The remaining sequences matched midgut ESTs to different extents: 19 sequences (1.59%) had E-values between 0 and 10^-150^, 20 sequences (1.67 %) had E-values of 10^-150 ^and 10^-100^, 24 sequences (2.01%) had E-values of 10^-100 ^and 10^-50^, 31 sequences (2.59%) had E-values of 10^-50 ^and 10^-20^, and 76 sequences (6.36%) had E-values of 10^-20 ^and 10^-5^. A total of 937 sequences (78.4%) had no hits with the available midgut ESTs.

In addition, we compared our unique sequences with those of the silkworm *B. mori*. We used BLASTN to search the all *B. mori *EST sequences available using a BLAST search site [14], given that the genome sequence is not fully annotated. A total of 492 from the 1,195 unique sequences (41.17%) had hits with silkworm sequences at E < 10^-5 ^(Table 6). Of these, 133 unique ESTs (27% of the 492 sequences) had E-values between 10^-100 ^and 10^-50^. A total of 703 sequences (58.8%) had no matches with silkworm sequences.

### Conserved *S. frugiperda *and *Drosophila *gene sequences

We found 11 highly conserved sequences between *S. frugiperda *and *Drosophila *genes based on BLASTX analyses. All of the 11 sequences were from contigs, with one, contig 134, having an E-value of 10^-154^. Six sequences had matches with their homologous *Drosophila *genes at an E-value of 0.

**Figure 2:**
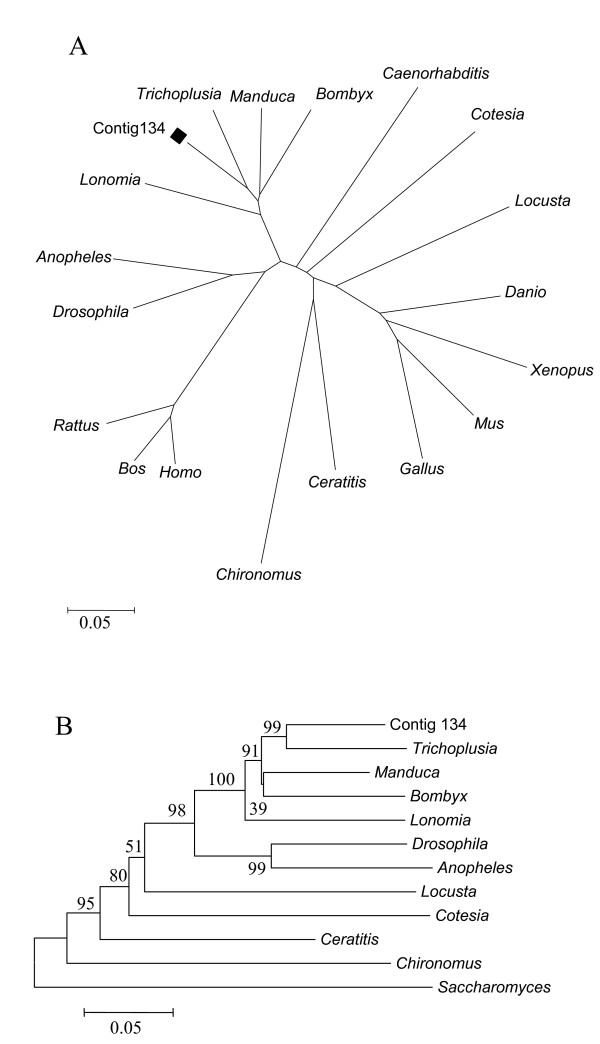
**Phylogenetic analysis with neighbor-joining tree**. A. The heat shock proteins of 10 insects and 8 other organisms (*Cotesia rubecula, Ceratitis capitata, Chironomus tentans, Manduca sexta, Locusta migratoria, Drosophila melanogaster, Anopheles gambiae, Lonomia oblique, Bombyx mori, Trichoplusia ni, Bos taurus*, *Gallus gallus, Rattus norvegicus, Danio rerio, Xenopus laevis, Caenorhabditis elegans, Mus musculus*, *Homo sapiens*) along with Contig 134 (heat shock 70 cognate 4 protein) are presented in unrooted phylogenetic analyses. B. Phylogenetic tree showing heat shock proteins with *Saccharomyces cerevisiae *as the outgroup. The bootstrap values (percentages) are indicated at the corresponding node.

We chose contig 134 for phylogenetic analysis given it was the most conserved sequence between *Spodoptera *and *Drosophila*. The sequences from heat shock 70 cognate 4 proteins were aligned with CLUSTALW and only similar sequences with complete coding sequences (CDS) were included in the alignment as described in Methods. Alignments of the heat shock protein 70 cognate 4 (contig 134) with similar ones in the Class Insecta (Fig. 2) showed that the heat shock protein 70 cognate 4 of *S. frugiperda *formed a single clade with *Trichoplusia ni, Manduca sexta, Bombyx mori*, and *Lonomia oblique*, as expected since all these organisms belong to the order Lepidoptera. This clade shares a common ancestor with members of other orders, Diptera, Orthoptera, and Hymenoptera, and with insects in other clades (*e.g*., *Ceratitis capitata*,*Chironomus tentans*, *Drosophila melanogaster*, *Anopheles gambiae*, *Locusta migratoria*, and *Cotesia rubecula)*.

### Functional classification of *S. frugiperda *ESTs

Gene Ontology (GO) has been widely used to characterize gene function annotation and classification [15]. GO describes gene function using controlled vocabulary and hierarchy including molecular function, biological processes, and cellular communication. In this report, we used well annotated GO information of *Drosophila melanogaster *to interpret the gene function of our ESTs. Each unique sequence from *S. frugiperda *was assigned the same gene function of the best BLASTX hit (E ≤ 10^-5^) with *Drosophila *sequences based on the annotated GO of *Drosophila *[15]. This method has been successfully used to annotate bee brain EST function [16].

The major GO categories for the unique sequences included those outlining gene molecular function (Additional file 1- Table 7), biological processes (Additional file 2- Table 8), and cellular components (Additional file 3- Table 9). The highest final child GO term for molecular function was the hydrogen transporting two-sector ATPase in the nucleotide binding category. The highest final GO term for biological processes was protein biosynthesis, under the protein metabolism and biosynthesis categories, which had 84 unique sequences accounting for 7% of the total unique sequences matched in this category. The largest number for a final GO term in cellular components was cytosolic large ribosomal subunit under both the ribosome and cytosol categories. Seventy-eight unique sequences belonged to this GO term, which accounted for 6.5% of the total unique sequences annotated for cellular components.

We found 13 unique sequences (1.1%) showing significant similarity with *Drosophila *signal transduction factors (Table 3). Among these, 6 sequences belonged to the receptor binding category and the remaining 7 sequences belonged to receptor and receptor signaling proteins.

Based on GO, we also found one sequence for an apoptosis-related gene, pyes2-ct_017_g10.p1ca, which showed similarity to the Dros *ophila Aac11 *gene. Two additional sequences, pb42ad-1_001_f09.pb42 primer and pyes2-ct_010_g11.p1ca, showed significant similarity to *Drosophila Gnbp3*, a gene involved in defense and immunity.

### Pathway analysis based on the Kyoto Encyclopedia of Genes and Genomes (KEGG) classification

KEGG has been widely used for pathway mapping [17]. Enzyme commission (EC) numbers were used to judge which sequences pertained to a specific pathway. Twenty-nine unique sequences including 8 contigs and 21 singlets accounted for 2.4% of unique sequences and matched enzymes with an EC number. Within these 29 sequences, 11 unique sequences (1% of total) containing 4 contigs and 7 singlets were mapped to KEGG biochemical pathways (Table 4). Genes involved in amino acid metabolism had the highest mapping sequences (5) and 6 mappings. Genes involved in carbohydrate metabolism included 5 sequences and 8 mappings. Other genes included those involved in nucleotide metabolism (2 sequences), translation (2 sequences), energy metabolism (1 sequence), lipid metabolism (1 sequence), and metabolism of other amino acids (1 sequence). Contigs 35 and 97 each had 4 mappings to different pathways under a different metabolism category, which was the highest number for a single sequence. Contig 120, contig 152, pyes2-ct_008_e11.p1ca, and pyes2-ct_012_c04.p1ca mapped to two pathways. The remaining pathway-assigned sequences only mapped to one pathway.

### EST database

**Figure 3:**
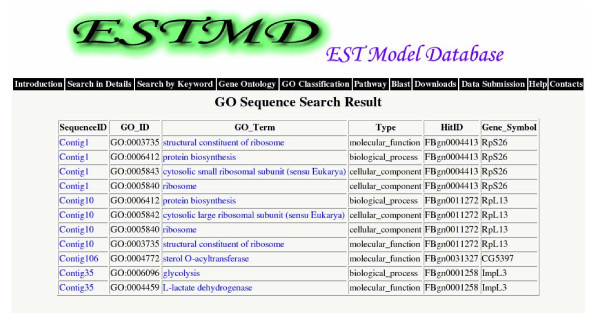
**Gene Ontology search results**. The search results used a sequence file by choosing all three ontologies.

To efficiently manage and retrieve information in the ESTs analyzed in this project, we developed an EST model database (ESTMD version 1) [9]. The EST model database is a highly efficient, web-accessible, user-friendly relational database. It provides several tools to search raw, cleaned, and assembled EST sequences, genes and GO, as well as pathway information. The user may input and submit keywords or IDs to the server using the web interface. ESTs and annotated function data are in the relational database and results are sent back to the user in proper formats in response to a query. It also provides a clear contig view and BLAST searches, data submission, and download pages. In both the GO and KEGG pathway search pages, ESTMD allows users to search GO and pathways not only by single gene name, symbol, or ID, but also by using a file that contains a batch of sequence IDs or FlyBase IDs. All the sequence function classification based on GO and KEGG pathway in this study was done using ESTMD. The file search provision in ESTMD allows users to obtain information regarding the possible function of many ESTs or genes at one time instead of searching them individually (Fig. 3).

## Discussion

Single-pass sequencing was performed on 3,365 cDNA clones derived from two SF-21 cDNA libraries. Using this sequence data, we have established an EST database comprised of 1,195 unique sequences from the SF-21 cell line, derived from the lepidopteran insect *S. frugiperda*. A total of 667 unique sequences (57%) had homology to sequences found in *Drosophila*. These sequences will be useful for comparative genomics within and outside the Lepidoptera, establishing microarrays, and as probes to either clone or down-regulate genes of interest by RNA interference in order to perform studies related to *Spodoptera*, other closely related Lepidoptera, or their pathogens.

This is one of two published annotated EST studies available for *S. frugiperda*. A prior report included a similar scale project as that reported here with 5,937 ESTs of which 1,855 were unique sequences obtained from a clonally-derived cell line of SF-21, Sf9 [12]. The majority of unique sequences in the previous study consisted of the highly abundant ribosomal protein genes and these were found to have low codon usage bias [12]. Our data provides 776 novel *S. frugiperda *sequences. A small percentage of our sequences (20.2%) had similarities, whether these reflect cell line specific differences is not clear at this time. Together these two studies and other available *S. frugiperda *ESTs constitute seminal work on the genome sequence of *S. frugiperda*. The sequences reported in this study have been made available for incorporation into Spodobase [18].

Many insects within the Lepidoptera, including the fall armyworm *S. frugiperda *(family Noctuidae), are pests that cause significant annual damage to a number of field crops and tree foliage worldwide. Deciphering their genomic sequences will aid in developing improved pest control agents, such as baculoviruses and polydnaviruses/parasitic wasps. Although these pathogens are being used or sought as biological control agents, there remains ample room for improvement of their entomopathogenic properties.

Finally, molecular tools have been used in the study of Lepidoptera or have been derived from Lepidoptera. The transposable element *piggyBac *was discovered in the lepidopteran *T. ni *(cabbage looper) and has been used to create somatic and germline transformations in a number of organisms including crickets, butterflies, *Plasmodium falciparum*, and more recently, mice [19-22]. Lepidoptera are also amenable to down-regulation of genes by RNA interference and transgenic techniques [23]. Thus, knowledge of the genomics of Lepidoptera will aid in their manipulation or use as molecular tools.

## Conclusion

We have established an EST database from the *S. frugiperda*-derived cell line SF-21, containing 1,195 unique sequences. Lepidoptera are among the most diverse insects and as such, sequences and EST databases from various genomes will be instrumental in assessing species-specific genes, phylogeny, and parallels within species of the same order. In addition, comparative analyses with available genomes of other insects including *A. mellifera*, *D. melanogaster*, *A. gambiae*, *Ae. aegypti*, and *T. castaneum *will yield additional insights since these include members of distinct orders (Hymenoptera, Diptera, and Coleoptera), providing a more accurate picture of the conserved pathways and the order-specific gene elements in the Insecta.

## Methods

### cDNA library construction

Two independently constructed cDNA libraries were used for sequencing. For both libraries, mRNA isolated from log phase SF-21 cells was used for cDNA synthesis, and the libraries were directionally cloned into plasmid vectors. One library was custom made by Clontech using the plasmid vector pB42AD. The pB42AD library had a titer of 3.6 × 10^13 ^colony forming units per ml. The second library was constructed using the SuperScript™ Plasmid System (Invitrogen) and the plasmid vector pYES2/CT (Invitrogen) that had been modified by addition of a *Sal *I linker at the *Bam *HI site. The pYES2/CT library had a titer of 1.2 × 10^12 ^colony forming units per ml. The average insert size for both libraries was 1.5 kbp.

### EST sequencing

Initially, approximately 200 randomly selected clones from each library were subjected to single-pass sequencing using 5' vector primers. DNA sequencing was performed by MWG Biotech (High Point, NC). Although both libraries yielded acceptable sequence quality, the pYES2/CT library appeared to yield slightly longer sequences; consequently, the remainder of the sequencing was performed using clones from the pYES2/CT library.

### Sequence processing

Sequence information was stored in chromatograph trace files, and Phred [24] was used to perform Base-calling [24]. Flanking vector and adaptor sequences were trimmed using Cross-match [25] and Lucy [26], while low quality bases (quality score < 20) were cleaned at both sequence ends by using our custom program. RepeatMasker [27] was used to mask repeated sequences, and the masked sequences were further screened to remove contaminating sequences from bacteria and viruses using BLASTN [13]. High quality ESTs were assembled using CAP 3 [7] and verified with Phrap [8], which perform similar tasks. After assembly, Consed [24] was used to assess contig quality, and assembled ESTs were chosen for further analysis. Contigs flagged for possible miss-assembly were manually edited in Consed and potential chimeric ESTs or other suspect ESTs were removed from the pool of traces.

### Sequence annotation

High quality assembled ESTs were annotated using BLASTX through NCBI and our local BLAST server. We searched several databases including the NCBI non-redundant and *Drosophila *protein databases. The BLAST results were automatically extracted and transferred into a relational database. The sequences reported in this study (2,367 ESTs) have been deposited in GenBank under accession numbers [GenBank: DY792773 to DY795139].

### Functional classification

Functional classification of unique sequences from *S. frugiperda *was based on GO [15]. Unique sequences, including contigs and non-overlapping singlets, were used to search *Drosophila *predicted protein databases using BLASTX. The *Drosophila *genes corresponding to the best hits at a threshold of E-value ≤ 10^-5 ^with known GO term were assigned to the query "*Spodoptera *sequences". All the matched GO information was stored in our local MySQL database.

### Pathway assignments

Pathway assignments were carried out according to KEGG mapping [17]. EC numbers [28] were assigned to unique sequences that had BLASTX scores with a cut off value of E = 10^-5 ^or less upon searching SWIR protein databases. The sequences were mapped to KEGG biochemical pathways according to the EC distribution in the pathway database.

### Phylogenetic analysis

Proteins were aligned with CLUSTALW using only CDS in the alignment. The alignment was then used to generate phylogenetic trees by the Neighbour-Joining method using the MEGA version 2.1 program. The bootstrap values for the nodes were determined by analyzing 500 bootstrap replicate data sets to estimate the strength of the groupings.

### Database implementation

A web-based interface of the database was created using HTML and JavaScript to evaluate the validation of the input on the client side and to reduce the burden on the server side. Apache 2.0 was used as the http web server, while Tomcat 4.1 was the servlet container. Both of these programs were developed and maintained on UNIX, Linux, and Windows NT, ensuring that ESTMD was transplantable and platform-independent. ESTMD is currently hosted on Red Hat 9, and it can be implemented in MySQL 4.0 or higher version. The main tables were on clones, ESTs, uniSequence, uniHit, FlyBase and FlyBaseDetails. The server-side programs were implemented by Java technologies. Servlet and JavaServer Pages were used to communicate between users and databases and to implement a query. XML and XSLT technologies were used to describe, generate, and express GO trees.

## Authors' contributions

ALP and RJC contributed to the conception and design of the study, coordinated the study, and were involved in drafting the manuscript. YDeng participated in data analysis and drafting of the manuscript. YDong and VT were involved in data analysis. All authors read and approved the final manuscript.

## Supplementary Material

Additional file 1- Table 7 Table 7. Distribution of molecular functional categories based on gene ontology for *Spodoptera frugiperda *unique sequences Click here for file

Additional file 2- Table 8 Table 8. Distribution of biological processes based on gene ontology for *Spodoptera frugiperda *unique sequences Click here for file

Additional file 3- Table 9 Table 9. Distribution of cellular component categories based on gene ontology for *Spodoptera frugiperda *unique sequences Click here for file

## Figures and Tables

**Table 1:** Most abundantly represented transcripts in the *Spodoptera frugiperda *cDNA library.

**Contig**	**ESTs**	**GI#**	**Bit score**	**E-value**	**Identities**	**Gene descriptions**	**Organism**
Contig 190	13	40363707	238	1e-61	129/215	cytochrome oxidase II	*Glyphodes bicolor*
Contig 98	13	18314310	241	5e-96	122/159	Cytochrome c oxidase subunit 3	*Ostrinia furnacalis*
Contig 116	13	16566722	458	e-128	229/244	ribosomal protein S3A	*S. frugiperda*
Contig 146	13	18253045	138	5e-32	76/112	60s acidic ribosomal protein P2	*S. frugiperda*
Contig 61	14	7302066	249	6e-65	129/243	CG11522-PB, isoform B	*D. melanogaster*
Contig 225	14	18253043	147	1e-34	78/111	60s acidic ribosomal protein P1	*S. frugiperda*
Contig 70	15	54609281	452	e-126	233/307	ribosomal protein SA	*B. mori*
Contig 139	17	27260896	411	e-113	204/218	ribosomal protein S2	*S. frugiperda*
Contig 160	17	22094837	389	e-106	199/283	Cytochrome b	*Samia cynthia ricini*
Contig 23	18	18253041	550	e-155	283/315	60 Saccadic ribosomal protein PO	*S. frugiperda*
Contig 134	21	12585261	1119	0	568/608	Heat shock 70 kDa cognate 4	*Manduca sexta*
Contig 239	30	39752635	480	0	234/241	elongation factor-1 alpha F2	*D. melanogaster*
Contig 141	46	1438928	685	0	364/504	Cytochrome oxidase subunit 1	*Feltia jaculifera*
Contig 19	63	552886	226	e-111	102/104	ND-1 protein gene	*S. frugiperda*

**Table 2:** Comparative analysis of *Spodoptera *ESTs to *Drosophila*^1 ^and other sequences.

	**All matches**						***Drosophila***					
	**Contig**		**Singlets**		**Total**		**Contig**		**Singlets**		**Total**	
**Homology**	**N**	**%**	**N**	**%**	**N**	**%**	**N**	**%**	**N**	**%**	**N**	**%**
E ≤ 10 ^-150^	11	5	0	0	11	2	10	5	0	0	10	1
E ≤ 10 ^-100^	30	15	23	4	53	7	30	16	23	5	53	8
E ≤ 10 ^-50^	92	45	191	37	283	39	88	46	186	38	274	40
E ≤ 10 ^-20^	48	24	189	36	237	33	45	24	179	37	224	33
E ≤ 10 ^-5^	22	11	118	23	140	20	17	9	99	20	116	17
Total matched	203	91	521	55	724	61	190	85	487	50	677	57
No match	41	9	430	45	471	39	54	22	484	50	518	43
**Total**	**244**	**100**	**951**	**100**	**1195**	**100**	**244**	**100**	**951**	**100**	**1195**	**100**

^1 ^[11]

**Table 3:** Signal transduction sequences of *Spodoptera frugiperda *compared with *Drosophila *genes.

***S. frugiperda *** **sequences**	**Flybase number**	**Hit length**	**Bit score**	**E-value**	**Identities**	***Drosophila* gene**	**Gene description**
pyes2-ct_019_b03.p1ca	FBgn0039541	836	275	1.00e-74	138/266	*Cg12876*	Signal transduction activity
pyes2-ct_006_f12.p1ca	FBgn0035771	753	360	e-104	164/231	*Cg8583*	Signal recognition particle binding
pyes2-ct_021_a12.p1ca	FBgn0027363	689	84	1.00e-17	35/59	*Stam*	Signal transducing adaptor molecule
Contig 14	FBgn0003963	1191	139	2.00e-33	86/223	*ush*	Involved in torso signaling pathway
pyes2-ct_005_g01.p1ca	FBgn0035771	753	222	1.00e-58	104/149	*cg8583*	Involved in signal recognition particle complex
pyes2-ct_006_f09.p1ca	FBgn0037277	2228	314	3.00e-86	152/242	*Cg17735*	Ligand-dependent nuclear receptor interactor activity
pyes2-ct_028_g06.p1ca	FBgn0020618	318	199	1.00e-51	96/107	*Rack1*	Receptor of activated protein kinase C 1
Contig 140	FBgn0020618	318	578	e-165	274/319	*Rack1*	Receptor of activated protein kinase C 1
pyes2-ct_030_g06.p1ca	FBgn0004569	444	67	4.00e-12	29/43	*argos*	Receptor antagonist activity
p42ad_2_001_b07.p1cb.exp	FBgn0037113	1258	133	3.00e-32	71/131	*cg33291*	Putative protein binding
pyes2-ct_003_e12.p1ca	FBgn0013984	2144	73	1.00e-13	74/313	*InR*	Insulin like receptor
Contig 220	FBgn0031547	406	125	3.00e-43	65/191	*CG3212*	Scavenger receptor activity involved in defense response
Contig 226	FBgn0037357	773	102	7.00e-25	52/62	*sec23*	Putative GTPase activator activity

**Table 4:** KEGG pathway mapping for *Spodoptera frugiperda *unique sequences.

**KEGG pathway**	**Sequence ID**	**Number of sequences**	**Percentage of total**
Carbohydrate metabolism		4	36
Glycolysis/gluconeogenesis	Contig 35, contig 97, pyes2-ct_008_e11.p1ca	3	27
Fructose and mannose metabolism	pyes2-ct_027_b04.p1ca	1	9
Pyruvate metabolism	Contig 35, contig 97	2	18
Propanoate metabolism	Contig 35	1	9
Starch and sucrose metabolism	pyes2-ct_010_g11.p1ca	1	9
Energy metabolism		1	9
Carbon fixation	Contig 97	1	9
Lipid metabolism		1	9
Sphingoglycolipid metabolism	pyes2-ct_010_a06.p1ca	1	9
Nucleotide metabolism		2	18
Purine metabolism	Contig 97, contig 120	2	18
Amino acid metabolism		5	45
Alanine and aspartate metabolism	Contig 120	1	9
Arginine and proline metabolism	pyes2-ct_026_h11.p1ca	1	9
Glycine, serine and threonine metabolism	pyes2-ct_012_c04.p1ca	1	9
Cysteine metabolism	Contig 35	1	9
Phenylalanine, tyrosine and tryptophan biosynthesis	Contig 152, pyes2-ct_008_e11.p1ca	2	18
Metabolism of other amino acids		1	9
Selenoamino acid metabolism	pyes2-ct_021_f10.p1ca	1	9
Translation		2	18
Aminoacyl-tRNA biosynthesis	Contig 152, pyes2-ct_012_c04.p1ca	2	18

**Table 5:** Comparative analysis of *Spodoptera frugiperda *SF-21-derived ESTs with Sf9- and midgut-derived ESTs^1^.

	***S. frugiperda* Sf9ESTs**						***S. frugiperda* midgutESTs**					
	**Contig**		**Singlets**		**Total**		**Contig**		**Singlets**		**Total**	
**Homology**	**N**	**%**	**N**	**%**	**N**	**%**	**N**	**%**	**N**	**%**	**N**	**%**
E ≤ 0	243	99.59	176	18.51	419	35.06	7	2.87	81	8.52	88	7.36
0 < E ≤ 10^-150^	0	0.00	37	3.89	37	3.10	2	0.82	17	1.79	19	1.59
10^-150 ^< E ≤ 10^-100^	0	0.00	47	4.94	47	3.93	4	1.64	16	1.68	20	1.67
10^-100 ^< E ≤ 10^-50^	0	0.00	58	6.10	58	4.85	0	0.00	24	2.52	24	2.01
10^-50 ^< E ≤ 10^-20^	0	0.00	49	5.15	49	4.10	1	0.41	30	3.15	31	2.59
10^-20 ^< E ≤ 10^-5^	0	0.00	50	5.26	50	4.18	4	1.64	72	7.57	76	6.36
Total matched	243	99.59	417	43.85	660	55.23	18	7.38	240	25.24	258	21.59
No match	1	0.41	534	56.15	535	44.77	226	92.62	711	74.76	937	78.41
Total	**244**	**100**	**951**	**100**	**1195**	**100**	**244**	**100**	**951**	**100**	**1195**	**100**

^1 ^SF-21 ESTs, this report; Sf9 ESTs [12]; midgut ESTs (NCBI dbEST)

**Table 6:** Comparative analysis of *Spodoptera frugiperda *SF-21 ESTs with silkworm ESTs.

	***Bombyx mori* ESTs**					
	**Contig**		**Singlets**		**Total**	
**Homology**	**N**	**%**	**N**	**%**	**N**	**%**

E ≤ 0	53	21.72	7	0.74	60	5.02
0 < E ≤ 10^-150^	17	6.97	12	1.26	29	2.43
10^-150 ^< E ≤ 10^-100^	51	20.90	60	6.31	111	9.29
10^-100 ^< E ≤ 10^-50^	44	18.03	89	9.36	133	11.13
10^-50 ^< E ≤ 10^-20^	13	5.33	79	8.31	92	7.70
10^-20 ^< E ≤ 10^-5^	13	5.33	54	5.68	67	5.61
Total matched	191	78.28	301	31.65	492	41.17
No match	53	21.72	650	68.35	703	58.83

Total	**244**	**100**	**951**	**100**	**1195**	**100**

^1 ^SF-21 ESTs, this report; Sf9 ESTs [12]; midgut ESTs (NCBI dbEST)
