# Supplementary material for: Analysis and functional annotation of expressed sequence tags from the fall armyworm Spodoptera frugiperda
Source: BMC Genomics. 2006 Oct 19;7:264. doi: 10.1186/1471-2164-7-264 (PMC1634997; doi:10.1186/1471-2164-7-264)
Supplement: Additional file 1- Table 7 — Table 7. Distribution of molecular functional categories based on gene ontology for Spodoptera frugiperda unique sequences [file 1471-2164-7-264-S1.pdf]

Table 7. Distribution of molecular functional categories based on gene ontology for *Spodoptera frugiperda* unique sequences

Gene Ontology term	*S. frugiperda*	
	**Unique sequences**	**Percentage of total**
Enzyme	305	56
Hydrolase	115	21
Oxidoreductase	44	8
Transferase	44	8
Kinase	19	3
Helicase	17	3
Lyase	11	2
Ligase	11	2
Isomerase	9	2
Small protein conjugating enzyme	3	1
Binding	266	49
Nucleic acid binding	116	21
DNA binding	47	9
Transcription factor	14	3
RNA binding	43	8
Translation factor	25	5
Nucleotide binding	53	10
Protein binding	34	6
Calcium binding	5	1
Lipid binding	2	<1
Heavy metal binding	2	<1
Selenium binding	1	<1
Structure molecule	93	17
Structure constituent of ribosome	81	15
Structure constituent of cytoskeleton	10	2
Transporter	81	15
Carrier	48	9
Ion transporter	15	3
Channel/pore	5	1
Electron transporter	3	<1
Intracellular transporter	3	<1
Drug transporter	1	<1
Transcription regulator	46	8
Transcription factor	22	4
Transcription cofactor	5	1
Signal transducer	13	2
Receptor signal protein	3	<1
Receptor	4	<1
Receptor binding	6	1
Chaperone regulator	30	6
Translation regulator	25	5
Enzyme regulator	8	1
Motor	6	1
Protein tagging	5	1
Cell adhesion molecule	5	1
Antioxidant	2	<1
Apoptosis regulator	2	<1
Defense/immunity protein	2	<1
