# Supplementary material for: Analysis and functional annotation of expressed sequence tags from the fall armyworm Spodoptera frugiperda
Source: BMC Genomics. 2006 Oct 19;7:264. doi: 10.1186/1471-2164-7-264 (PMC1634997; doi:10.1186/1471-2164-7-264)
Supplement: Additional file 2- Table 8 — Table 8. Distribution of biological processes based on gene ontology for Spodoptera frugiperda unique sequences [file 1471-2164-7-264-S2.pdf]

Table 8. Distribution of biological processes based on gene ontology for *Spodoptera frugiperda* unique sequences

Gene Ontology term	*S. frugiperda*	
	**Unique sequences**	**Percentage of total**
Cell growth and/or maintenance	361	68
Metabolism	303	57
Protein metabolism and modification	185	35
Biosynthesis	125	24
Nucleic acid metabolism	51	10
DNA metabolism	23	4
RNA metabolism	13	2
Catabolism	33	6
Phosphorus metabolism	13	2
Alcohol metabolism	10	2
Amino acid metabolism	4	1
Carbohydrate metabolism	4	1
Lipid metabolism	3	1
Oxygen metabolism	2	<1
Pigment metabolism	1	<1
Electron metabolism	1	<1
One-carbon compound metabolism	1	<1
Transport	49	9
Cell organization and biogenesis	49	9
Response to stress	19	4
Cell cycle	16	3
Cell growth	4	1
Cell proliferation	3	1
Cell morphogenesis	2	<1
Homeostasis	1	<1
Cell motility	1	<1
Development	78	15
Morphogenesis	46	9
Embryonic development	23	5
Reproduction	17	3
Sex determination	5	1
Pattern specification	5	1
Cell differentiation	4	1
Growth	2	<1
Larval development	2	<1
Regulation of gene expression	1	<1
Cell fate commitment	1	<1
Aging	1	<1
Cell communication	70	13
Response to external stimulus	21	4
Cell-cell signaling	17	3
Signal transduction	12	2
Cell recognition	2	<1
Cell adhesion	1	<1
Behavior	14	3
Physiological processes	4	1
Death	2	<1
