# Supplementary material for: Analysis and functional annotation of expressed sequence tags from the fall armyworm Spodoptera frugiperda
Source: BMC Genomics. 2006 Oct 19;7:264. doi: 10.1186/1471-2164-7-264 (PMC1634997; doi:10.1186/1471-2164-7-264)
Supplement: Additional file 3- Table 9 — Table 9. Distribution of cellular component categories based on gene ontology for Spodoptera frugiperda unique sequences [file 1471-2164-7-264-S3.pdf]

**Table 9. Distribution of cellular component categories based on gene ontology for *Spodoptera frugiperda* unique sequences**

| Gene Ontology term | *S. frugiperda* Unique sequences | Percentage of total |
|---|---|---|
| Cell | 420 | 93 |
| Intracellular | 357 | 79 |
| Cytoplasm | 302 | 67 |
| Cytosol | 148 | 33 |
| Ribosome | 127 | 28 |
| Mitochondrion | 72 | 16 |
| Cytoskeleton | 26 | 6 |
| Endoplasmic reticulum | 7 | 2 |
| Golgi apparatus | 4 | 1 |
| Vacuole | 3 | 1 |
| Endosome | 2 | < 1 |
| Nascent complex | 1 | < 1 |
| Nucleus | 85 | 19 |
| Ribonucleoprotein complex | 21 | 5 |
| Chromosome | 8 | 2 |
| Cell cortex | 2 | < 1 |
| Membrane | 94 | 21 |
| Mitochondrial membrane | 32 | 7 |
| Inner membrane | 27 | 6 |
| Plasma membrane | 19 | 4 |
| Integral to membrane | 10 | 2 |
| Endomembrane | 2 | < 1 |
| Cell fraction | 1 | < 1 |
| Extracellular | 19 | 4 |
| Unlocalized | 13 | 3 |
